# Simultaneous Deletion of p21^Cip1/Waf1^ and Caspase-3 Accelerates Proliferation and Partially Rescues the Differentiation Defects of Caspase-3 Deficient Hematopoietic Stem Cells

**DOI:** 10.1371/journal.pone.0109266

**Published:** 2014-10-06

**Authors:** Carmen Carrillo García, Tamara Riedt, Jin Li, Manuela Dotten, Peter Brossart, Viktor Janzen

**Affiliations:** Department of Internal Medicine III, Division of Hematology/Oncology, University of Bonn, Bonn, Germany; Emory University, United States of America

## Abstract

Specialized blood cells are generated through the entire life of an organism by differentiation of a small number of hematopoietic stem cells (HSC). There are strictly regulated mechanisms assuring a constant and controlled production of mature blood cells. Although such mechanisms are not completely understood, some factors regulating cell cycle and differentiation have been identified. We have previously shown that Caspase-3 is an important regulator of HSC homeostasis and cytokine responsiveness. p21^cip1/waf1^ is a known cell cycle regulator, however its role in stem cell homeostasis seems to be limited. Several reports indicate interactions between p21^cip1/waf1^ and Caspase-3 in a cell type dependent manner. Here we studied the impact of simultaneous depletion of both factors on HSC homeostasis. Depletion of both Caspase-3 and p21^cip1/waf1^ resulted in an even more pronounced increase in the frequency of hematopoietic stem and progenitor cells. In addition, simultaneous deletion of both genes revealed a further increase of cell proliferation compared to single knock-outs and WT control mice, while apoptosis or self-renewal ability were not affected in any of the genotypes. Upon transplantation, p21^cip1/waf1-/-^ bone marrow did not reveal significant alterations in engraftment of lethally irradiated mice, while Caspase-3 deficient HSPC displayed a significant reduction of blood cell production. However, when both p21^cip1/waf1^ and Caspase-3 were eliminated this differentiation defect caused by Caspase-3 deficiency was abrogated.

## Introduction

In mammals, mature blood cells are produced over the entire lifetime of an organism. This process is tightly regulated in order to maintain a supply of mature blood cells and avoid HSC exhaustion and at the same time to prevent malignancies. Thus, mechanisms strictly controlling differentiation and self-renewal of hematopoietic stem and progenitor cells (HSPCs) are critical. Nevertheless, the exact molecular mechanisms regulating HSC (or HSPC) biology are still not fully understood.

We have previously demonstrated the relevance of Caspase-3 in the regulation of hematopoietic stem cells [1]. Although the importance of Caspase-3 is undisputed in apoptosis, we found no detectable changes in the rate of apoptosis within the hematopoietic stem cell population in vivo. Instead, the proliferation of hematopoietic stem cells was significantly accelerated and the ability to differentiate into multiple cell lines reduced. Hereby Caspase-3 was found to regulate the proliferation of primitive hematopoietic cells by modulating their responsiveness to cytokines and thus selectively restraining specific signaling pathways to maintain stem cell quiescence. Similar effects in differentiation were also observed in other cell systems such as neuronal, osteogenic and myogenic stem cells [2]–[4]. However, cell cycle activity is influenced in distinct cell systems in different ways. For example, deletion of Caspase-3 in osteoblasts causes a deceleration of their proliferation rate [4] whereas in splenic B lymphocytes Caspase-3 deficiency leads to hyperproliferation [5].

In recent years, several molecular mechanisms that affect proliferation, differentiation and self renewal of stem cells have been defined. In the adult organism, under physiological conditions, hematopoietic stem cells are found mostly in a quiescence state [6]–[8]. The cell cycle progression in stem cells, as in other cells, is regulated by the strict control of interactions between cyclins, cyclin dependent kinases (CDK) and their inhibitors (CDKI). The importance of CDKIs for the proliferation and repopulation ability of hematopoietic stem cells has been extensively studied [9]–[16].

There are conflicting reports on the importance of p21^Cip1/Waf1^ for cell cycle regulation and self-renewal capacity in hematopoietic stem cells. On the one hand, p21^Cip1/Waf1^ deletion was found to promote HSC proliferation, resulting in an increase in their absolute number under steady state conditions. On the other hand, p21^Cip1/Waf1-/-^ stem cells showed an impairment in self-renewal and thus a faster exhaustion in the context of serial transplantation. These results thus suggested that p21^Cip1/Waf1^ is a key molecule that governs the entry of HSC into the cell cycle [9]. However in a more recent study, no differences in proliferation or self-renewal under steady-state conditions were observed between WT and p21^Cip1/Waf1-/-^ hematopoietic stem cells, albeit using a different approach to analyze cell cycling, suggesting a limited effect of p21^Cip1/Waf1^ in HSC in steady state conditions. Nevertheless, an impaired response to cellular stress survival was detectable in the p21^Cip1/Waf1-/-^ HSC, suggesting a more important function under stress conditions [15], [17]. These differences may be attributed to the use of different mouse strains as well as of different approaches to investigate HSC biology in both studies. Therefore, a precise role of p21^Cip1/Waf1^ in hematopoietic stem cells is not quite clearly defined.

All biological processes are regulated by a complex web of interactions between proteins within different signaling pathways. The deletion of a single protein can often be diminished by compensation mechanisms, masking the physiological role of the single molecule. Simultaneous deletion of several molecules that interact or have additive effect can be helpful in uncovering the roles of individual proteins more precisely. For example, deletion of p21^Cip1/Waf1^ alone does not affect the aging of cells or the life span of mice [15], however the simultaneous deletion with telomerase (Terc) causes an improvement in the repopulation ability and self-renewal of hematopoietic cells as well as an extension of the life span of the mice [12].

p21^Cip1/Waf1^ is a transcriptional target of the tumor suppressor gene p53 and plays an important role in the induction of cell cycle arrest in the context of DNA damage. Depending on the extent of the cell defect the damage will be repaired or cell death will be initiated [18]. Depending on the cell type and the conditional circumstance of the cells, p21^Cip1/Waf1^ can exert either a pro- or an anti-apoptotic effect [19]. For example, in Hep2G cell lines, the formation of a p21^Cip1/Waf1^/Caspase-3 complex has been described to inhibit pro-Caspase-3 cleavage site and thus activation of Caspase-3, resulting in prevention of apoptosis [20]–[22]. Furthermore, it has been reported that an inhibition of apoptosis by ionizing radiation in p21^Cip1/Waf1-/-^ colon carcinoma cells, in which no p21^Cip1/Waf1^-Caspase-3 direct association was observed, was due to a blockade of Caspase-9 and ultimately also Caspase-3 activation [23].

On the other hand in Caspase-3 deficient cells, a change in the p21^Cip1/Waf1^ expression or activity of the cyclin dependent kinases has been described. In splenic B lymphocytes Caspase-3 has been shown to be responsible for p21^Cip1/Waf1^ cleavage, thus in Caspase-3^-/-^ lymphocytes uncleaved p21^Cip1/Waf1^ accumulates promoting hyperproliferation. Interestingly, in Caspase-3/p21^Cip1/Waf1^ double knockout mice, the hyperproliferation of B-lymphocytes was significantly attenuated compared to Caspase-3^-/-^ cells [5]. In contrast to lymphocytes, an increased expression of p21^Cip1/Waf1^ has been shown in Caspase-3 deficient osteoblasts, resulting in cell cycle arrest and replicative senescence [4]. All this, together with the elevated p21^Cip1/Waf1^ mRNA expression levels observed in HSC cells [9] suggest the possibility of a regulatory interaction between Caspase-3 and p21^Cip1/Waf1^ in hematopoietic stem cells.

Therefore, in the present study we aimed to analyze the impact of a simultaneous deletion of p21^Cip1/Waf1^ and Caspase-3 on the apoptosis, expansion, proliferation, differentiation and self-renewal of hematopoietic stem cells. We found that the frequency of apoptotic events as well as the ability to sequentially reconstitute lethally irradiated mice was not altered in HSCs lacking both Caspase-3 and p21^Cip1/Waf1^ or corresponding cells lacking Caspase-3 or p21^Cip1/Waf1^ alone. However, the proliferation of the primitive hematopoietic cells lacking both factors was substantially accelerated compared with single KO or control HSPCs. Furthermore, the additional deletion of p21^cip1/waf1^ was able to at least partially rescue the differentiation defect observed in Caspase-3 mutant bone marrow cells.

## Materials and Methods

### Mice

Caspase-3^-/-^ [5], [1] and p21^Cip1/Waf1-/-^ [9], [15] mice were kept at the animal facility from the University of Bonn (Haus für Experimentelle Therapie) according to institutional guidelines. Mice were sacrificed by increasing CO_2_ concentration as indicated by the correspondent authorities. All animal experiments were approved by the Federal office for Nature, Environment and Consumer protection North Rhine Westphalia (Landesamt für Natur, Umwelt und Verbraucherschutz NRW) and the local authorities at the University of Bonn.

Both mice colonies were interbred and heterozygous parents were mated to obtain mice mutant for Caspase-3 and p21^Cip1/Waf1^ (e.g. DKO). Both colonies were backcrossed on a C57BL/6 and CD45.1 background.

### Automated peripheral blood analysis

Peripheral blood was obtained by tail vein nicking, collected in EDTA-coated containers (Microtainers, BD Biosciences) and measured within a few hours after collection. Total numbers of white blood cells, erythrocytes and platelets were obtained using the Hemavet 950 Analyzer from Drew Scientific (Dallas, USA).

### Sample collection and flow cytometric analysis

#### Blood

From anti-coagulated whole blood, erythrocytes were lysed and the remaining cells were fixed with FACS Lysing Buffer (BD Biosciences) according to manufacturer's instructions. After washing with staining buffer (SB, PBS containing 1% fetal calf serum Gold, FBS, PAA Laboratories GmbH) the blood cells were stained for surface markers expressed on mature blood cells, in particular B220-APC-eF780 for B-lymphocytes, CD3e-PE-Cy7 for T-lymphocytes, Gr1-eF660 for Granulocytes, and Mac1α-eF450 for monocytes. For congenic strain discrimination, anti-CD45.1-PE and anti-CD45.2-FITC antibodies were used. All antibodies were purchased from eBioscience (San Diego, USA) and reactivity was against mouse, unless stated otherwise.

#### Bone marrow

Bone marrow was freshly prepared from the hind legs of a sacrificed mouse and washed with SB. Then the cells were stained with the antibody panel as indicated below. Flow cytometry was performed in a BD FACS Canto II flow cytometer (BD Biosciences). Briefly, the samples were resuspended in SB; cells were centrifuged and the pellet was resuspended in the corresponding antibody mixture and incubated at room temperature or 4°C for 20 minutes. The excess of antibody was washed by addition of 1ml of SB to each sample. Then, the samples were centrifuged; cells were resuspended with the adequate volume of SB and measured by FACS.

To identify the hematopoietic stem cell containing populations, maturating bone marrow cells were excluded by staining with a cocktail of biotinylated antimouse antibodies to Mac-1a (CD11b), Gr-1 (Ly-6G and Ly-6C), Ter119 (Ly-76), CD3 and B220 (CD45R) with subsequent staining with streptavidin conjugated with eF450 or PerCP. Additionally, c-Kit-APC, Sca1-PE-Cy7 (Ly 6A/E), CD150-AF488 (BioLegend, San Diego, USA) and CD48-PE, were used to discriminate the different bone marrow cell populations.

To assess cell cycle in the primitive population, bone marrow cells were stained with lineage antibodies and c-Kit, Sca-1, CD150 and CD48, as described above, as well as Ki-67 or BrdU. To stain for Ki-67-FITC or BrdU-APC cells were fixed and permeabilized using the BrdU Flow Kit (BD Biosciences) following the manufacturer's instructions. Nuclei were counterstained with DAPI (1∶2000, Molecular Probes). For BrdU incorporation we used the APC-BrdU Flow Kit (BD Biosciences) following a single intraperitoneal injection of BrdU (2 mg/animal) and an additional administration of 1 mg/ml of BrdU (Sigma) mixed to drinking water for approximately 12 or 24 hours, as indicated.

For the apoptosis assay we used the above described surface markers staining combined with AnnexinV-FITC (BD Biosciences) and counterstained with DAPI (1∶2000, Molecular Probes) to asses cell viability.

### Transplantation Assays

For bone marrow transplantation, 2×10^6^ whole bone-marrow cells from 8- to 12-week-old WT and mutant (Caspase-3^-/-^, p21^Cip1/Waf1-/-^ and DKO) CD45.1 mice were injected into lethally irradiated (9.5 Gy as a split dose) female recipient C57BL/6 CD45.2 mice, obtained from Charles River laboratories. Engraftment efficiency in recipients has been monitored by donor contribution of CD45.1-positive cells using FACS analysis. Peripheral blood cell analyses were performed at different time points post-transplantation. Sixteen weeks post-transplantation, recipients were used as donors for the next transplantation cycle and for assessment for the contribution of donor-derived primitive bone marrow subpopulations. Transplants were discontinued after the second or third round of transplantation.

For the competitive repopulation assay, 1×10^6^ whole bone marrow cells from 8–12 weeks old WT CD45.1&CD45.2 mice were mixed with the same amount of whole bone-marrow cells from age paired CD45.1 WT or mutants (Caspase-3^-/-^, p21^Cip1/Waf1-/-^ or DKO) and injected into lethally irradiated 8 weeks old CD45.2 recipient mice. Repopulation was assessed by flow cytometry at different time points after transplant for multilineage reconstitution as indicated.

### Cytokine stimulation and detection of protein phosphorylation

Bone marrow cells from different animals were harvested and pre-cultured in medium (X-Vivo15, Lonza) without cytokines for 10 minutes and stimulated with cytokines (stem cell factor, SCF at 1 ng/ml, R&D Systems; and thrombopoietin, TPO, at 4 ng/ml, R&D Systems) for the time indicated. Subsequently, the cells were fixed and permeabilized using Phosflow Lyse/Fix and Perm/Wash buffers (BD Biosciences) according to manufacturer's instructions. Cells were then stained for surface markers to define the HSC-containing population as described above and with antibodies against pERK1/2-FITC (1∶20; Cell Signaling).

## Results

### Absence of both, Caspase-3 and p21^Cip1/Waf1^ has limited effect on peripheral blood cell production

**Figure 1:**
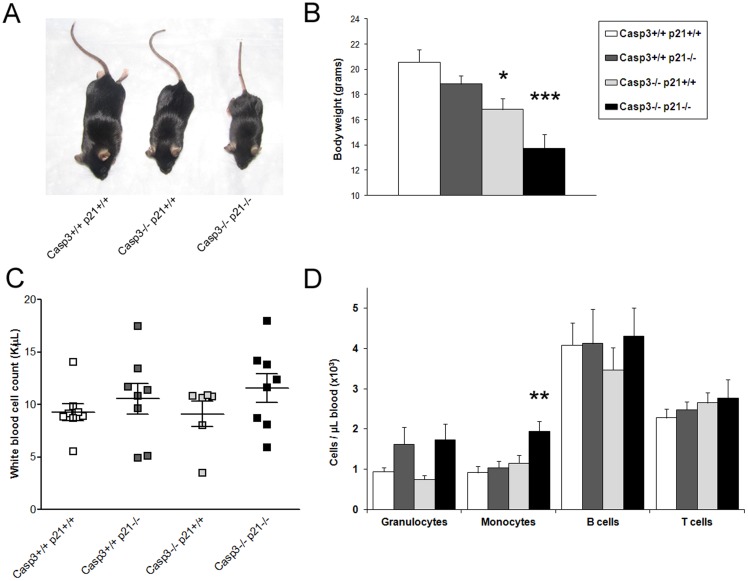
Depletion of Caspase-3 and p21^Cip1/Waf1^ minimally affect blood cell production in steady state. (A, B) Comparison of the weight of young mutants and WT counterpart: the DKO mice show a much more prominent reduction in body size than the already small Caspase-3^-/-^ mice. (C-D) Analysis of peripheral blood in steady state. (C) No significant differences were found in the white blood cells count of the different mutants. (D) Subtle changes were found in the different blood cell subpopulations by FACS analysis. Values are mean ± SEM; n≥3; *p≤0.05; ** p≤0.01.

In this study we have investigated the effect of the simultaneous deletion of two proteins, p21^Cip1/Waf1^ and Caspase-3, in the regulation of murine hematopoiesis. Although the role of both proteins as well as their interaction has been already described in other systems, the relevance of such interaction in homeostasis of the primitive hematopoietic compartment has not been studied yet. Hence, we have generated mice lacking both proteins p21^Cip1/Waf1^ and Caspase-3. Although Caspase-3 deficient mice are not born at mendelian ratio as previously reported [5],[24], animals lacking either Caspase-3 or p21^Cip1/Waf1^ were described to survive to a young adult stage (6–8 weeks old) [5], [15]. However, in our observation the double knockout mice (DKO) were not only rare, way far under the expected mendelian ratio, but had also a limited survival rate and only few reached an adult age. They showed a distinct reduction in body size compared to their littermates, being even smaller than the Caspase-3 deficient animals, which have been reported to exhibit a reduced size compared to WT littermates due to a delay in bone ossification [4] (Figure 1 A, B). We speculate that the smaller body size could result in an additional disadvantage at the pre-weaning period in the competition for pups nursing, eventually leading to the premature death of DKO pups.

First we analyzed whether the depletion of both proteins has any effect on the composition of mature blood cells. Peripheral blood analyses showed no differences in white, or red blood cells or thrombocyte counts compared to WT controls (Figure 1 C, and S1 A–B). However, FACS based differential analysis of the white blood cells of the Caspase-3/p21^Cip1/Waf1^ double mutants show an increased number of monocytes compared to WT (Figure 1 D). However, the biological relevance of such subtle change remains unclear. No significant differences were found in any of the other analyzed cell populations under steady state conditions.

### Simultaneous deletion of p21^Cip1/Waf1^ and Caspase-3 synergize to increase frequency of HSPC

**Figure 2:**
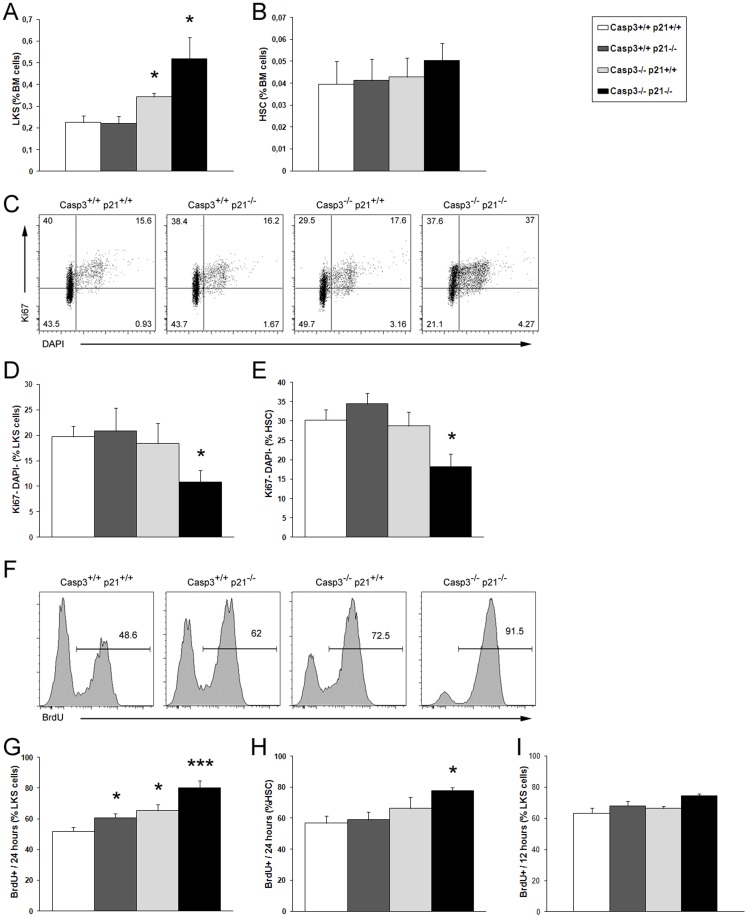
Increased HSPC population size and proliferation rate in the DKO mouse in steady state conditions. (A) The double mutant shows an additional enlarged LKS population than the already larger population observed in the Caspase-3^-/-^ mouse, compared to the WT and p21^Cip1/Waf1-/-^ counterparts. (B) No differences in the HSC populations of the different animals were found. (C) Representative dot plots of LKS cells stained with Ki67 and DAPI showing the augmented number of cycling cells in the DKO respect of the other mice. (D and E) Cell cycle analyses in different genotypes by Ki67 expression. Reduced proportion of DKO LKS cells (D) and HSC (E) in the quiescent state (Ki67- DAPI -). (F) Representative histograms showing BrdU incorporation in LKS cells. (G and H) The three mutant mice showed an elevated number of BrdU positive LKS cells (G) but only the DKO shows the same increase in HSC (H) after 24 hours pulse. (I) In the transplant setting, no differences in the proportion of cycling cells between the four genotypes were found after 12 hours BrdU pulse. Values are mean ± SEM; n≥3; *p≤0.05; ** p≤0.01; *** p≤0.001.

Next we aimed to investigate the frequency of the immature bone marrow compartment. Using the immunophenotypic approach to identify different subsets of hematopoietic stem and progenitor populations one can at least get a rough estimate on changes in the frequency of the most primitive bone marrow cells. Analysis of the primitive bone marrow compartment by flow cytometry revealed that depletion of both Caspase-3 and p21^Cip1/Waf1^ resulted in an even more pronounced increase in a population enriched in HSPC (lineage- cKit+ Sca1+; hereafter referred to as LKS cells) compared to the already augmented pool observed in the Caspase-3^-/-^ mice [1] (Figure 2 A). A detailed analysis of the subpopulations within the LKS cells showed that such difference in the LKS population size was not due to an increased pool of the most primitive hematopoietic cells (lineage-cKit+Sca1+CD48-CD150+; hereafter referred to as hematopoietic stem cells, HSC) (Figure 2 B) but to an augmented pool of multipotential progenitors (lineage-cKit+Sca1+CD48+CD150-; hereafter referred to as MPPs) (Figure S1 C). No difference was observed in the frequency of the myeloid restricted progenitor population (lineage-cKit+Sca-1-; hereafter referred to as progenitors) (data not shown).

Both Caspase-3 and p21^Cip1/Waf1^ are well-known apoptosis mediators. However, both molecules deploy their potential in apoptosis induction differently depending on cell type and homeostatic conditions. We have previously described that Caspase-3 has a limited effect on HSPC apoptosis in vivo [1]. However, no studies have been conducted so far to assess a possible role for p21^Cip1/Waf1^ in HSC apoptosis. Hence we studied here whether the depletion of p21^Cip1/Waf1^ alone or in combination with Caspase-3 would affect the susceptibility of primitive hematopoietic cells to apoptotic stimuli in vivo. Thus, we analyzed freshly isolated bone marrow cells for apoptotic events using the AnnexinV/DAPI assay. No significant differences in the proportion of apoptotic cells (AnnexinV+/DAPI-) were found in any population of the studied genotypes (Figure S1D and data not shown), confirming that neither Caspase-3 nor p21^Cip1/Waf1^ play a major role in HSC apoptosis under steady state conditions. However, we cannot exclude that under some conditions of forced apoptosis induction, like some cytotoxic drugs or gamma irradiation, one of the proteins may impact the frequency of cell death in vivo.

### Proliferation of HSPC is augmented in absence of Caspase-3 and p21^Cip1/Waf1^

Whereas Caspase-3 has been shown to be an important factor regulating HSPC cell cycle entry and its depletion results in an increased number of cycling HSPCs [1], the role of p21^Cip1/Waf1^ in HSPC proliferation has been controversial [9], [15], [25]. To readdress this issue we have utilized wild type mice as well as p21^Cip1/Waf1-/-^, Caspase-3^-/-^ and double mutant (DKO) animals, all in the C57/Bl6 background, and analyzed stem cell cycling with two different approaches in steady state condition and under proliferative stress of stem cell transplants. In some of the previous studies of the single mutant mice, cell cycle analyses were performed using the RNA-dye (Pyronin Y) that is temperature sensitive, changing the fluorescence intensity during data acquisition and thus might affect the results. Here we used a method that has been widely recognized as a reliable discriminator of resting (G0) and cycling (G1-S-G2-M) cells using intracellular measurement of Ki67 protein expression [26]–[29]. In addition, Cheng et al. [9], performed cell cycle analyses only in the lineage negative population, not in the HSC enriched LKS subset of cells.

First we analyzed the cell cycle status using the Ki67/DAPI approach in freshly isolated bone marrow under steady state conditions to determine the frequency of different cell subsets in different phases of the cell cycle as it has been previously described [26]–[29]. Ki67/DAPI staining within the LKS and HSC populations revealed a reduction in the percentage of cells in G0 phase of the cell cycle (Ki67- DAPI-) in the DKO (Figure 2 C-E), correlating to a higher percentage of cells in S-G2-M phase (Ki67+ DAPI+) (Figure S1 E, F), indicating hyperproliferation of LKS cells and HSC lacking both proteins. However, no detectable differences in the distribution of the cell cycle stages were detectable in p21^Cip1/Waf1^ or Caspase-3 single mutant hematopoietic precursor cells, using the Ki67/DAPI assay. As the stem cell compartment is a very infrequently cycling population in its nature, small but biologically still relevant differences may not be detectable by the Ki67/DAPI approach, as this method is only able to provide a snapshot measurement at a given time point of a frequently cycling population. To reveal such small differences we performed BrdU incorporation experiments. We determined the frequency of cells that have initiated at least one cell division within 24 hours of BrdU administration in the different populations of the single and double mutant animals. Using this approach we could demonstrate that both Caspase-3 and p21^Cip1/Waf1^ deficient HSPCs have undergone significantly more cell divisions than the wild type controls, and that DKO cells displayed an even more pronounced increase in the proliferation rate than the single mutants (Figure 2 F–H, compare DKO with Caspase-3^-/-^ and p21^Cip1/Waf1-/-^). However, these differences are more obvious in the LKS population while analyses of the HSC enriched cell subset revealed only significant accelerated proliferation in the DKO animals (Figure 2H). These data highlight, that deletion of both, Caspase-3 and p21^Cip1/Waf1^, not only affect cell cycling in the more restricted HSPC, but also accelerates proliferation in the most immature hematopoietic stem cells.

Next we sought to investigate whether the absence of one of the proteins or both would have an impact on proliferation of primitive hematopoietic cells under mitogenic stress conditions such as bone marrow transplant. Upon transplant of a limited number of stem cells in a lethally irradiated host stem cells need to undergo a multitude of cell divisions to restore the stem cell pool and at the same time provide downstream progenitors to reconstitute the animals with functioning blood cells. Hence, we have performed BrdU incorporation assay at different time points during the engraftment process after whole bone marrow transplantation (3, 5, and 9 weeks). Figure 2I shows exemplary results of BrdU incorporation in LKS cells at 5 weeks after the transplantation. Notably, as we expected the primitive hematopoietic compartment to cycle at a higher rate under transplant condition, we reduced the time of BrdU exposure to 12 h. In all genotypes the fraction of cells that has undergone cell division is considerably higher than the one observed in steady state condition confirming an accelerated proliferation in the transplant setting. However, at all time points we did not observe any significant differences in proliferation activity of LKS cells between the genotypes. We reason that under stress conditions the proliferative activity of hematopoietic stem and progenitor cells are cycling at maximum rate so the cell cycle control of Caspase-3 or p21^Cip1/Waf1^ is overridden.

Taken together, we conclude that under steady-state conditions the simultaneous deletion of Caspase-3 and p21^Cip1/Waf1^ results in an augmented proliferation compared to the effect of the individual mutations on HSPC, while under transplant conditions none of the molecules has an effect on HSPC proliferation.

### Upon transplantation, additional deletion of p21^Cip1/Waf1^ ameliorates the differentiation defects of Caspase-3 deficient cells

**Figure 3:**
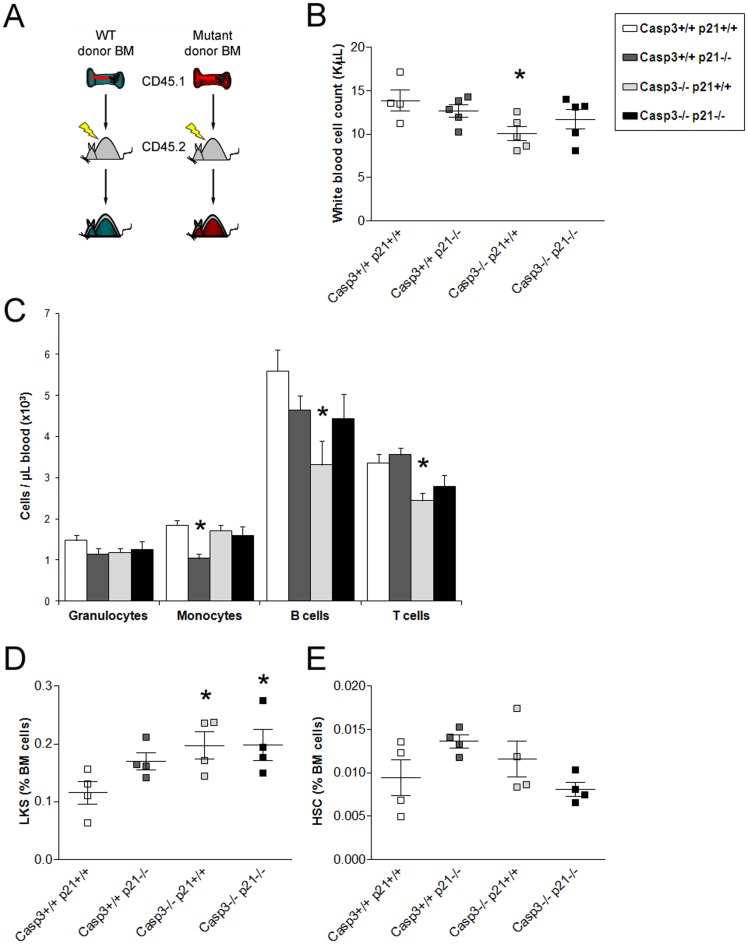
Simultaneous depletion of Caspase-3 and p21^Cip1/Waf1^ partially rescues the differentiation defect observed in the Caspase-3^-/-^ in a transplant setting. (A) Scheme of the whole bone marrow transplant setting. Test bone marrow from CD45.1 bearing WT, p21^Cip1/Waf1^, Caspase-3 or DKO bone marrow was transplanted into lethally irradiated 45.2 recipients and monitored for peripheral blood and bone marrow reconstitution. (B) Recipients from Caspase-3^-/-^ derived cells showed significantly fewer white blood cells count (WBC) compared to their WT counterpart 12 weeks after transplant. (C) Long-term analysis of peripheral blood cells at 12 weeks post transplant show that the additional depletion of p21^Cip1/Waf1^ rescues the defects caused by Caspase-3 deletion. (D–E) Bone marrow analysis 20 weeks post transplant revealed an increase in the frequency of LKS cells in the Caspase-3^-/-^ and DKO (D) but not in HSC (E). Values are mean ± SEM; n≥3; *p≤0.05.

To examine whether the simultaneous deletion of Caspase-3 and p21^Cip1/Waf1^ affect the behavior of HSPC under stress conditions, we transplanted bone marrow cells from the corresponding single mutants and DKO animals into lethally irradiated WT recipients and monitored their peripheral blood and bone marrow reconstitution. A congenic mouse system was utilized such that donor cells all expressed the panhematopoietic surface marker CD45.1 and were transplanted into CD45.2 allele-bearing hosts (Figure 3 A). In the analysis of the peripheral blood twelve weeks after transplant we did not find a significant difference in the number of leukocytes or their subsets (except monocytes) between recipients of p21^Cip1/Waf1-/-^ and wild type bone marrow cells (Figure 3 B, C), while in mice receiving Caspase-3-null bone marrow we observed a significant reduction in white blood cells, more specifically in lymphocytes as seen by a differential blood analysis using flow cytometry (Figure 3 B, C), in accordance to what we described before [1]. However, recipients of the DKO bone marrow showed no significant differences in total leukocytes or one of the cell subsets compared to the wild type recipients. Of note, we also observed an increase in the LKS population in the bone marrow of recipients from all mutant genotypes (Figure 3 D). Nevertheless, those differences were not due to changes in the HSC population but to the accumulation of multipotent progenitors (MPPs, data not shown). Next we investigated the ability of mutant cells to repopulate irradiated hosts in the presence of competing WT donor cells. When bone marrow cells from WT CD45.1 mice were transplanted with an equal number of WT CD45.1&2 competitors into CD45.2 recipient mice (Figure S3 A), cells from the different mutants showed no significant differences in LKS cells contribution 20 weeks after transplantation compared to the WT recipients (Figure S3 B). When we analyzed the contribution of transplanted test cells to the peripheral blood we found a significantly reduced blood cell production in all lineages from Caspase-3^-/-^ donor cells (Figure S3 C), again underscoring a known differentiation deficit in long term repopulating HSC (20 weeks after transplant) in absence of Caspase-3 [1], [5]. However, in contrast to Caspase-3 deficient bone marrow the contribution of p21^Cip1/Waf1-/-^ and DKO derived mature blood cells in myeloid and lymphoid lineages was equivalent to the one observed in WT mice except for a significantly higher number of B cells in both mutants (Figure S3 C). Thus, in vivo competitive repopulating ability of bone marrow cells was improved by p21^Cip1/Waf1^ deficiency and the depletions of p21^Cip1/Waf1^ and Caspase-3 simultaneously again rescued the differentiation impairment caused by Caspase-3 depletion.

### Depletion of Caspase-3, p21^Cip1/Waf1^ or both does not affect HSC self-renewal ability

**Figure 4:**
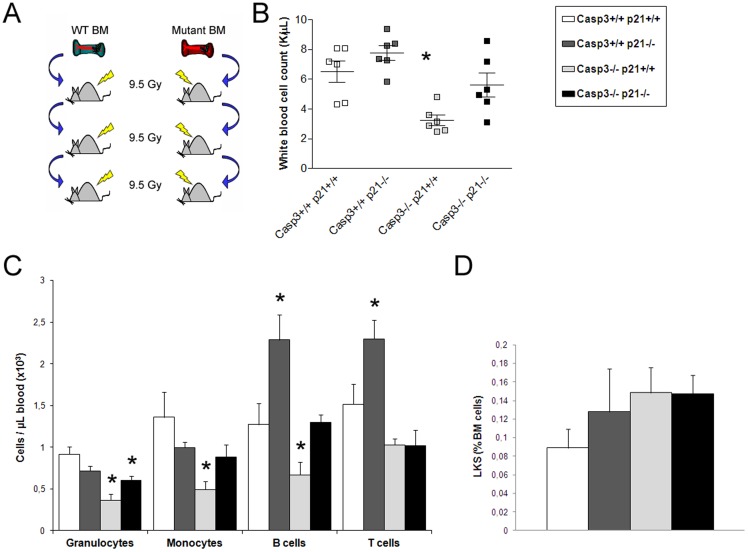
Self renewal ability of HSPC is not affected by Caspase-3 and/or p21^Cip1/Waf1^ deletion. (A) Scheme of the serial transplantation assay that was performed to analyze the ability of the different depleted HSCs to sustain the proliferative stress of repetitive transplantations rounds. (B) Analysis of peripheral blood counts 17 weeks after 2^nd^ round of bone marrow transplantation showed the same reduced white blood cell contribution of the Caspase-3^-/-^ as the one observed in the 1^st^ round of transplantation. (C) FACS analysis of peripheral blood cells 17 weeks after 2^nd^ round of bone marrow transplantation showed that simultaneous depletion of p21^Cip1/Waf1-/-^ rescues the effect of Caspase-3^-/-^ depletion in the different cell types. (D) No differences were found in the percentage of LKS cells in bone marrow 20 weeks after 2^nd^ round of bone marrow transplantation. Values are mean ± SEM; n≥3; *p≤0.05.

To analyze whether the deletion of both proteins Caspase-3 and p21^Cip1/Waf1^ has an impact on the stem cell self-renewal ability under conditions of proliferative stress, we performed sequential bone marrow transplantations. Bone marrow cells from each genotype (WT, Caspase-3^-/-^, p21^Cip1/Waf1-/-^ and DKO) were transplanted into lethally irradiated WT mice, and sixteen weeks after transplant two million bone marrow mononuclear cells from the transplanted recipients were used as donor cells for the next round of bone marrow transplantation. The same procedure was repeated for a third round (Figure 4 A). No difference in survival was noted in any group up to a third serial transplantation (data not shown). Additionally, bone marrow cells from all genotypes showed long term multilineage reconstitution without sign of exhaustion up to a 3^rd^ round of transplantation (Figure S2 A and data not shown).

Upon serial transplantation, a reduction in white blood cells in Caspase-3 transplanted animals was observed in both myeloid and lymphoid cells (Figure 4 B, C), consistent with previously reported impairment of Caspase-3 deficient stem cells to fully reconstitute lethally irradiated mice [1]. However, when p21^Cip1/Waf1^ was additionally deleted, the differentiation deficit of Caspase-3 deficiency was ameliorated. This rescue effect was not only observed in B-lymphocytes as it would be expected since p21^Cip1/Waf1^-Caspase-3 interaction has been previously postulated in splenic B-cells [5], but also in the myeloid cells (Figure 4 C). Analysis of the bone marrow compartment revealed no significant differences in the different bone marrow cell subpopulations between the genotypes. Especially the analyses of HSPC enriched LKS population at the end of the second cycle of bone marrow transplant revealed rather a tendency of preserved accumulation of LKS cells in Caspase-3^-/-^, p21^Cip1/Waf1-/-^ and DKO bone marrow recipients than loss of this primitive hematopoietic cell pool (Figure 4 D). These data again indicate that, neither in the single mutant nor in the double KO HSCs show signs of exhaustion even under highly proliferative conditions.

### p21^Cip1/Waf1^ deletion does not alter the sensitivity of Caspase-3 mutant LKS cells to exogenous signals

**Figure 5:**
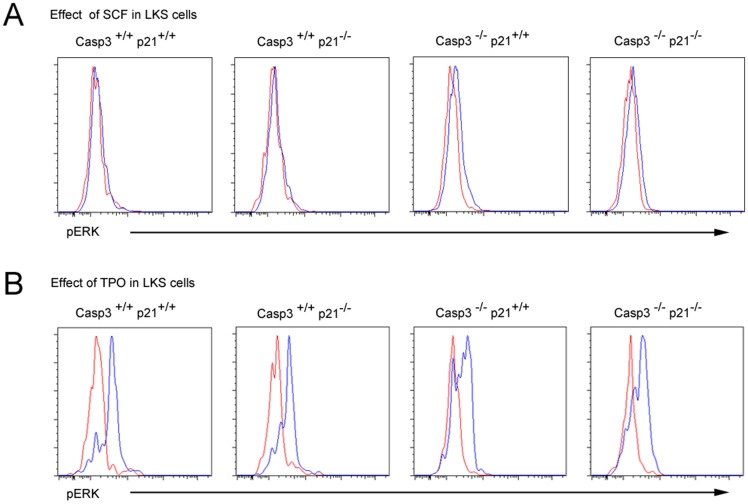
DKO LKS cells respond to cytokine stimulation in a manner resembling Caspase-3^-/-^ suggesting the involvement of a different mechanism in the rescue effect observed in hematopoiesis. (A) Stimulation of LKS cells with 1 ng/ml of SCF revealed and augmented signal transduction in the DKO similar to the one observed for the Caspase-3^-/-^. No difference was observed between the p21^Cip1/Waf1-/-^ and the WT mice. (B) Stimulation of LKS cells with 4 ng/ml TPO resulted in a decreased signal intensity in the DKO cells resembling those observed in the Caspase-3^-/-^ cells. No difference was observed between the p21^Cip1/Waf1-/-^ and the WT mice. FACS histograms are representative of at least three independent experiments.

We have previously reported that Caspase-3 modulates HPSC signaling in response to different cytokine stimulation [1]. We therefore sought to investigate whether p21^Cip1/Waf1^ is able to modulate the altered signaling pathways to rescue the phenotype observed in Caspase-3 deficient stem cells. To do this, isolated LKS cells from the DKO as well as the WT and single KO controls were exposed to SCF and TPO, cytokines that have been shown to affect HSC proliferation and where the signaling was impaired in absence of Caspase-3. Activation of ERK1/2 was examined as an analysis of the response to the cytokine stimulation. In absence of Caspase-3, elevated phosphorylation of ERK1/2 in LKS cells was noted compared with WT controls (Figure 5 A, B) in accordance with our previously report [1]. On the other hand, when stimulated with TPO Caspase-3^-/-^ LKS cells showed a decrease in ERK1/2 phosphorylation. LKS cells from DKO showed elevated activation of ERK equivalent to the one observed in Caspase-3^-/-^ derived cells (Figure 5 A) and reduced MAPK signaling in response to TPO (Figure 5 B). In the p21^Cip1/Waf1-/-^ cells, no difference in the response to SCF or TPO was observed compared to WT LKS cells (Figure 5 A, B). Taken together, our results suggest that the additional deletion of p21^Cip1/Waf1^ does not affect the response of HPSC to cytokines. Caspase-3 deletion results in increased sensitivity of primitive hematopoietic cells to cytokine induced cell signaling but the additional deletion of p21^Cip1/Waf1^ does not affect this sensitivity by neither reverting nor increasing it. Thus, we conclude that at the molecular level, LKS from the DKO show altered sensitivity to cytokine stimulation in a fashion that resembles the Caspase-3^-/-^ phenotype. Therefore, a different molecular mechanism than the one affecting the sensitivity to cytokines underlies the rescue effect of p21^Cip1/Waf1^ in Caspase-3 deficient hematopoiesis.

## Discussion

In the last years, many clues on the mechanisms that regulate HSC have been identified. Nevertheless, a complete understanding of HSC homeostasis is still far from being achieved. Preservation of HSC dormancy is crucial to maintain their function over the entire lifespan. However, there is also some controversy in the literature regarding the role of some cell cycle regulators in HSC function, as this applies to p21^Cip1/Waf1^. In the present study we confirm the previous assumption describing the importance of p21^Cip1/Waf1^ in regulating cell proliferation. However the impact of p21^Cip1/Waf1^ deletion on HSC on cycling is only detectable in a larger time frame and therefore not detectable using a snap-shot assay like Ki67/DAPI. We have previously identified Caspase-3 as an important factor regulating HSC quiescence by controlling their response to cytokines. In splenocytes Caspase-3 deficiency also resulted in a shift toward increased cell cycling [5]. However, the hyperproliferation of splenic B-cells was abolished by the simultaneous depletion of p21^Cip1/Waf1^, suggesting a close relation between Caspase-3 and p21^Cip1/Waf1^ in cell cycle regulation [5]. In the present study we confirm the previous reports indicating an increased cycling activity of HSPCs in absence of Caspase-3 or p21^Cip1/Waf1^. However, unlike in the splenic B-cells, the simultaneous deletion of both Caspase-3 and p21^Cip1/Waf1^ did not ameliorate, but rather resulted in even more pronounced proliferation of HSPCs, indicating a different mode of action of the cell cycle regulator p21^Cip1/Waf1^ in the immature hematopoietic compared to the further differentiated cells analyzed in the above mentioned studies.

There is also some evidence indicating a direct interaction of Caspase-3 and p21^Cip1/Waf1^ influencing their expression level and activity. Some studies have described a direct binding of p21^Cip1/Waf1^ and pro-Caspase-3 forming a complex that inhibits pro-Caspase-3 activation, acting then as a mechanism to protect cells from apoptosis [30]. On the other hand, it has been shown that p21^Cip1/Waf1^ is cleaved by Caspase-3, being this a prerequisite for cell death, converting cancer cells from growth arrest to undergoing apoptosis through activation of CDK2 [31], [32]. In addition, p21^Cip1/Waf1^ is known to block activation of the initiation Caspases-8 and -10 through inhibition of their cleavage, suppressing apoptosis and enhancing then cell survival [33]. In some cell types, although an interaction was expected, only a relation of the pathways was observed such as in colorectal carcinoma cells (HCT116) [23]. In light of those studies describing direct and indirect interactions of Caspase-3 and p21^Cip1/Waf1^, and because of the observed expression of p21^Cip1/Waf1^ in HSPC [9], [34] we investigated the effect of simultaneous deletion of these two molecules on HSPC homeostasis.

Under homeostatic conditions, adult HSCs are largely quiescent. Regulation of the balance between quiescence and cell cycle entry is believed to be critical for HSCs' biological role to prevent excessive proliferation and eventually exhaustion of the stem cell pool. p21^Cip1/Waf1^ is one of the key molecules in the regulation of cell cycle entry. Although initially p21^Cip1/Waf1^ was reported to be an important regulator of normal HSC function [9], more recent studies suggest that the role of p21^Cip1/Waf1^ is mainly restricted to genotoxically stressed HSCs without obvious role in cell cycle regulation [15], [25], [35]. Using a different experimental approach utilizing BrdU-incorporation assay, our data confirm the initially postulated role of p21^Cip1/Waf1^ in HSPC cycling. Interestingly, when the cycling activity of HSPCs was analyzed under conditions of proliferative stress of a bone marrow transplant, no differences in proliferation rate was detectable between the genotypes. We assume that the primitive hematopoietic cells are cycling at the maximum rate under transplant condition in the attempt to restore the stem cell pool, so neither Caspase-3 nor p21^Cip1/Waf1^ is able to suppress the proliferation drive.

The role of p21^Cip1/Waf1^ in different biological settings has been even more apparent when another molecule was eliminated in addition to p21^Cip1/Waf1^ deletion. Thus, p21^Cip1/Waf1^ has been shown to contribute to HSC regulation in a context of simultaneous depletion with p57^kip2^ resulting in an enhanced phenotype of the p57^ kip2^ depletion alone [25]. However, it has also been shown that although p57^ kip2^ plays a predominant role in HSC cell cycle regulation among Cip/Kip family of CKD inhibitors, it is the total abundance of CKIs of the Cip/Kip family that is important to determine HSC stemness [25]. In another study, deletion of p21^Cip1/Waf1^ in mice in which a telomerase (Terc) was deleted and thus displayed dysfunctional telomeres led to functional rescue of hematopoietic stem cells [12].

We have examined here the possible interaction of Caspase-3 and p21 in the hematopoietic system and its physiological importance in HSC homeostasis. In contrast to what was observed in splenocytes, p21^Cip1/Waf1^ deletion in addition to Caspase-3 deficiency does not reverse the augmented proliferation of Caspase-3^-/-^ HSPC but on the contrary additionally accelerated cell cycling. The increased proliferation was also associated with significant increase of the primitive cell number based on immunophenotypic examination of bone marrow subsets.

Earlier reports often associated increased proliferation of hematopoietic stem cells with faster exhaustion of the stem cell pool in serial bone marrow transplant experiments [13], [36], [37]. However, as reported previously by van Os and colleagues [15], we found no evidence for reduced self-renewal ability of p21^Cip1/Waf1^ deficient HSCs. In addition, when Caspase-3 was also simultaneously deleted, hematopoietic engraftment was not different compared to HSC from wild type animals despite an even more pronounced proliferation activity of the HSPCs. These data are in concordance with some other reports where increased proliferation did not necessarily affect self-renewal ability [1], [10], [16], [26].

We have previously reported that Caspase-3 is involved in the differentiation process at an early stage of hematopoiesis, leading to the reduction of different hematopoietic lineages upon transplantation [1]. p21^Cip1/Waf1^ deletion by itself does not display any impact on the differentiation process. However, when both molecules were simultaneously eliminated we detected a restored number of peripheral blood cells in all lineages. As we have observed, that the mutant animals displayed an accumulation of multipotent progenitors, one could speculate that the most pronounced increase in MPP cells in the double mutant animals could be responsible for the rescue of the differentiation defect observed in the double mutant mice. However, it is very hard to experimentally prove this hypothesis or exclude the possibility that increased number of MPPs are able to compensate the differentiation block.

A rescue effect of a phenotype in mutant mice caused by additional p21^Cip1/Waf1^ deletion was reported previously on different occasions. In telomerase deficient mice with markedly impaired stem cell function and short lifespan of the animals, additional deletion of p21^Cip1/Waf1^ results in improvement in stem cell maintenance and prolonged survival [12]. In splenic B cells lacking Caspase-3 and causing hyperproliferation of splenocytes, additional deletion of p21^Cip1/Waf1^ ameliorated the cycling activity [5]. Hence we speculate that Caspase-3 deletion leads to activation of p21^Cip1/Waf1^ dependent checkpoints during the hematopoietic maturation process resulting in a differentiation lag. However, we did not have the experimental possibility to further prove that at the molecular level. Nevertheless, we wondered whether p21^Cip1/Waf1^ deletion would also have an impact on sensitivity of HSPCs to exogenous signals. We found that p21^Cip1/Waf1^ by itself did not impair the intracellular signal intensity upon cytokine stimulation. Analyzing cells deficient for both Caspase-3 and p21^Cip1/Waf1^ revealed a similar signaling intensity as in Caspase-3 deficient cells. Thus, we conclude that the rescue effect of additional p21^Cip1/Waf1^ depletion in the differentiation ability caused by Caspase-3 deficiency is most likely based on different molecular mechanisms, than cytokine sensitivity.

## Supporting Information

Figure S1 **Corresponding to steady state;** **Figure 1** **and** **2** **.** (A and B) No significant differences were observed in the red blood counts (A) or platelets (B) of the different mice in steady state. (C) Elevated percentage of MPPs in the bone marrow of Caspase-3^-/-^ and DKO compared to WT. (D) No differences were observed in apoptosis analyzed by the AnnexinV-DAPI assay in LKS cells in steady state. (E and F) Higher percentage of DKO LKS (E) and HSC (F) cycling cells measured as Ki67+. Values are mean ± SEM; n≥3; *p≤0.05; ** p≤0.01.
(TIF)

Figure S2 **Corresponding to serial transplantation;** **Figure 4** **.** The repopulation capacity of the different genotypes is maintained in serial transplantations. FACS analysis demonstrating the percentage of test cells (CD45.1) from each population 17 weeks after 2^nd^ round of transplantation. Values are mean ± SEM; n≥3; *p≤0.05.
(TIF)

Figure S3 **Competitive transplantation assay.** (A) Scheme of the competitive repopulation assay, which was performed to test the ability of mutant stem cells to compete against WT HSC. (B) An equivalent contribution of test cells to the HSPC compartment is observed in all genotypes in the competitive transplant setting 20 weeks after transplant. (C) Analysis of peripheral blood counts 20 weeks after competitive bone marrow transplantation showed a significantly lower contribution of the Caspase-3^-/-^ bone marrow to all lineages of mature blood cells in peripheral blood compared to WT; whereas p21^Cip1/Waf1-/-^ and DKO show an increased contribution in the B cell compartment. Values are mean ± SEM; n≥3; *p≤0.05; ** p≤0.01.
(TIF)
