# Supplementary figures and images for: Simultaneous Deletion of p21Cip1/Waf1 and Caspase-3 Accelerates Proliferation and Partially Rescues the Differentiation Defects of Caspase-3 Deficient Hematopoietic Stem Cells
Source: PLoS One. 2014 Oct 6;9(10):e109266. doi: 10.1371/journal.pone.0109266 (PMC4186822; doi:10.1371/journal.pone.0109266)

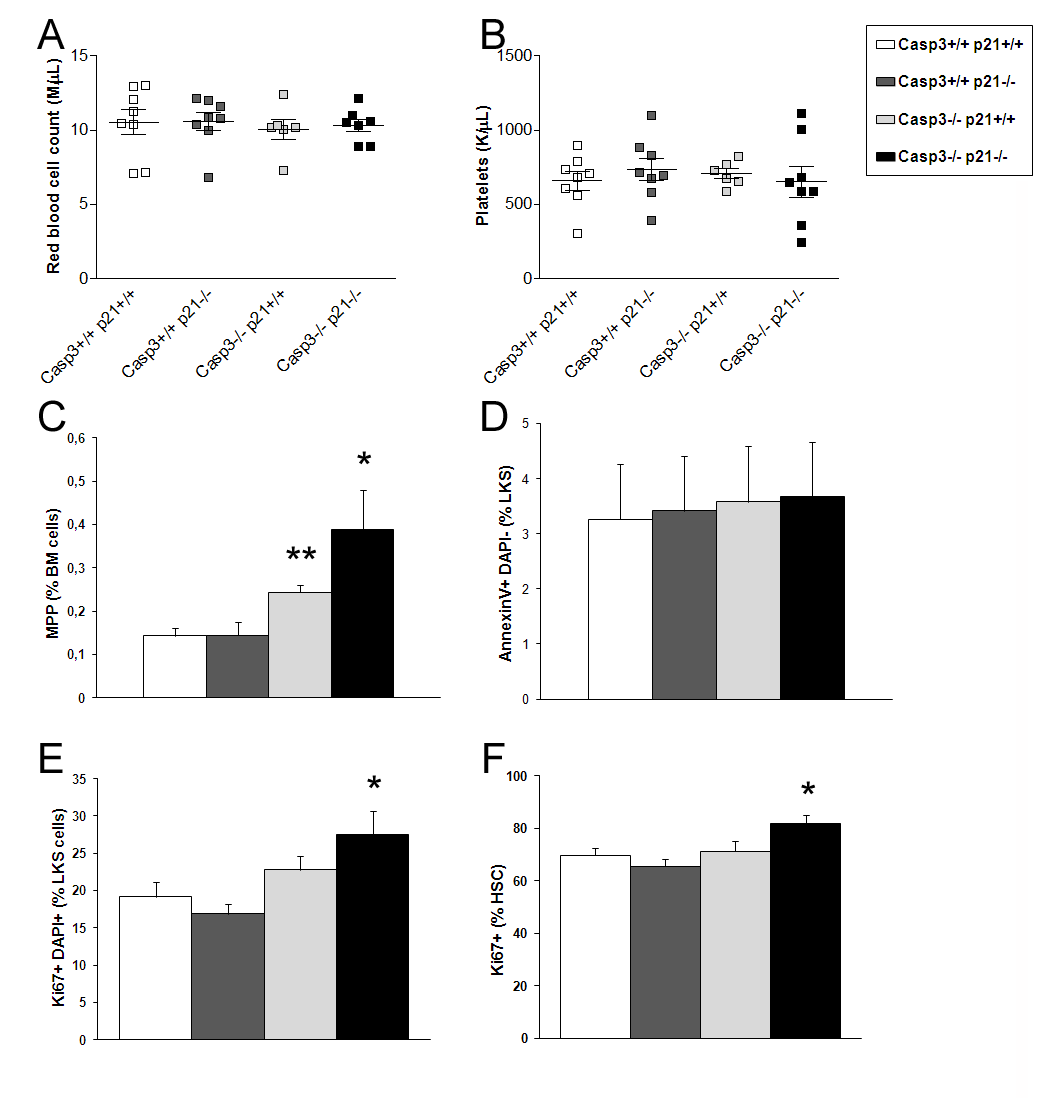

Supplement: Figure S1 — Corresponding to steady state; Figure 1 and 2 . (A and B) No significant differences were observed in the red blood counts (A) or platelets (B) of the different mice in steady state. (C) Elevated percentage of MPPs in the bone marrow of Caspase-3-/- and DKO compared to WT. (D) No differences were observed in apoptosis analyzed by the AnnexinV-DAPI assay in LKS cells in steady state. (E and F) Higher percentage of DKO LKS (E) and HSC (F) cycling cells measured as Ki67+. Values are mean ± SEM; n≥3; *p≤0.05; ** p≤0.01. (TIF) [file pone.0109266.s001.tif]

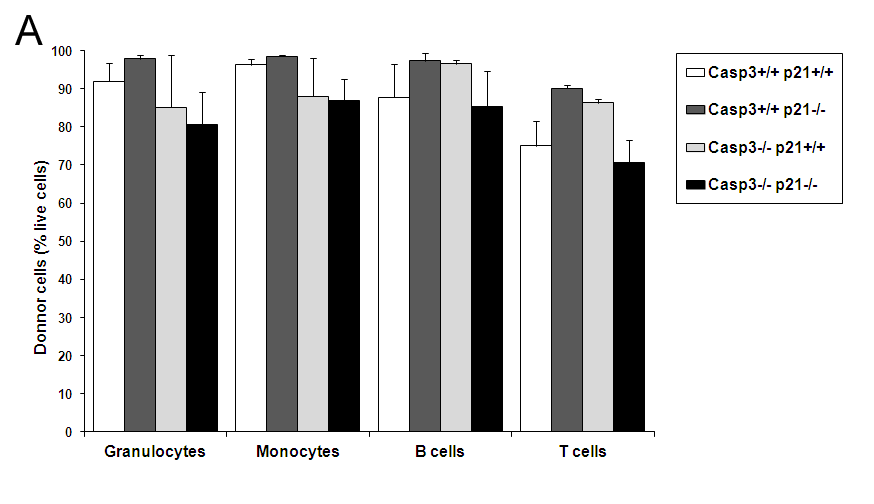

Supplement: Figure S2 — Corresponding to serial transplantation; Figure 4 . The repopulation capacity of the different genotypes is maintained in serial transplantations. FACS analysis demonstrating the percentage of test cells (CD45.1) from each population 17 weeks after 2nd round of transplantation. Values are mean ± SEM; n≥3; *p≤0.05. (TIF) [file pone.0109266.s002.tif]

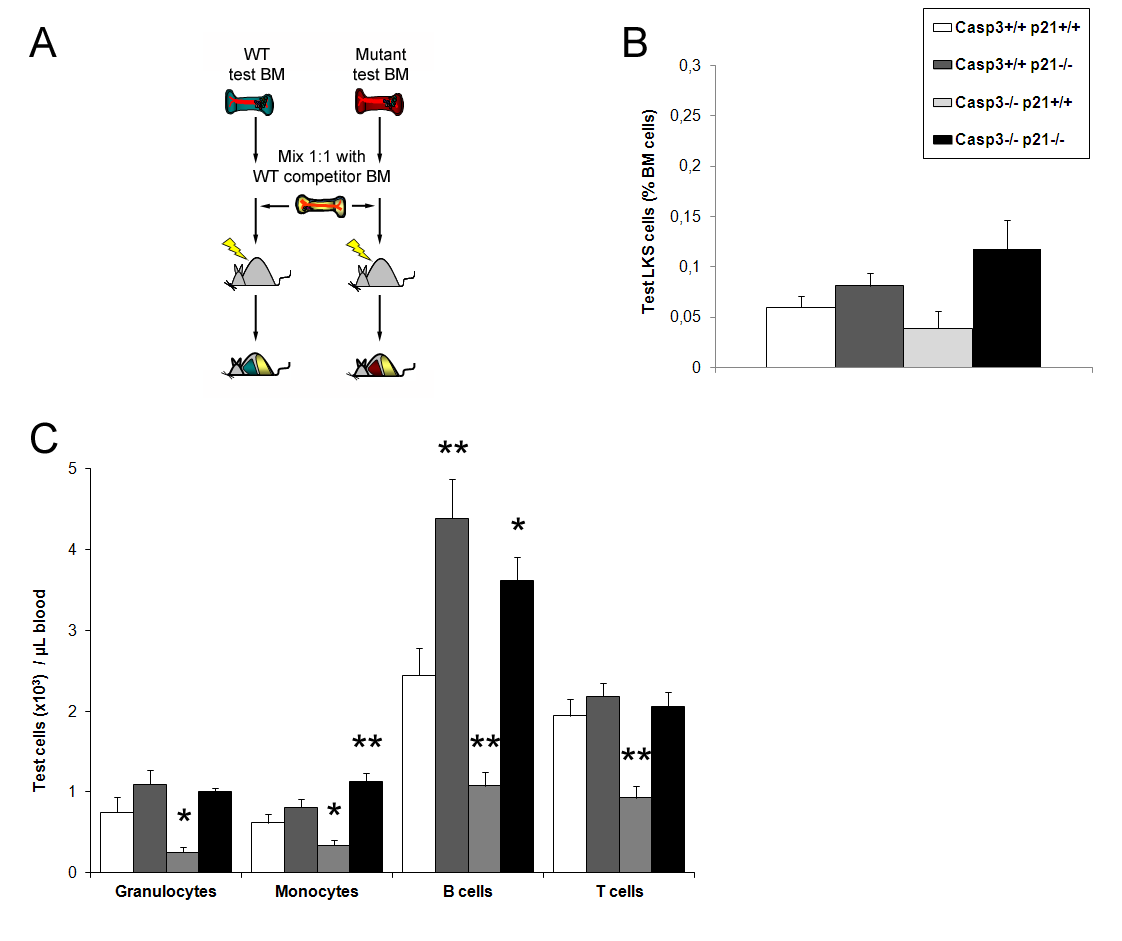

Supplement: Figure S3 — Competitive transplantation assay. (A) Scheme of the competitive repopulation assay, which was performed to test the ability of mutant stem cells to compete against WT HSC. (B) An equivalent contribution of test cells to the HSPC compartment is observed in all genotypes in the competitive transplant setting 20 weeks after transplant. (C) Analysis of peripheral blood counts 20 weeks after competitive bone marrow transplantation showed a significantly lower contribution of the Caspase-3-/- bone marrow to all lineages of mature blood cells in peripheral blood compared to WT; whereas p21Cip1/Waf1-/- and DKO show an increased contribution in the B cell compartment. Values are mean ± SEM; n≥3; *p≤0.05; ** p≤0.01. (TIF) [file pone.0109266.s003.tif]
